# Marked attenuation of P63 expression in plasmacytoid urothelial carcinoma with expanded immunohistochemical framework including E‐cadherin, P120, HER2 and TRPS1

**DOI:** 10.1111/his.70172

**Published:** 2026-05-05

**Authors:** Busra Yaprak Bayrak, Kemal Kosemehmetoglu, Busra Ozbek, Sercan Simsek, Murat Oktay, Ganime Coban, Dilara Irem Arslan Kahraman, Egemen Akıncıoglu, Melek Buyukgok, Selin Colak, Hatice Nese Dogan, Burak Gurel, Selva Kabul, Kerem Karaca, Elif Ozsagir, Ece Ozturk, Havva Serap Toru, Gupse Turan, Nazif Alperen Yıldırım, Ozlem Nur Yıldız, Yasemin Yuyucu Karabulut, Liang Cheng, Mahmut Akgul

**Affiliations:** ^1^ Department of Pathology, School of Medicine Kocaeli University Kocaeli Turkey; ^2^ Department of Pathology, School of Medicine Hacettepe University Ankara Turkey; ^3^ Department of Pathology Kutahya Evliya Celebi Research and Training Hospital Kutahya Turkey; ^4^ Department of Pathology Memorial Sisli Hospitals Group Istanbul Turkey; ^5^ Department of Pathology, School of Medicine Bezmialem Vakıf University Istanbul Turkey; ^6^ Department of Pathology Ankara Etlik City Hospital Ankara Turkey; ^7^ Department of Pathology Gulhane Research and Training Hospital Ankara Turkey; ^8^ Department of Pathology, School of Medicine Dokuz Eylul University Izmir Turkey; ^9^ Department of Pathology, School of Medicine Bursa Uludag University Bursa Turkey; ^10^ Department of Pathology, School of Medicine Akdeniz University Antalya Turkey; ^11^ Department of Pathology Sakarya Research and Training Hospital Sakarya Turkey; ^12^ Department of Pathology, School of Medicine Acibadem University Ankara Turkey; ^13^ Department of Pathology Kocaeli City Hospital Kocaeli Turkey; ^14^ Department of Pathology Kocaeli State Hospital Kocaeli Turkey; ^15^ Department of Pathology, School of Medicine Mersin University Mersin Turkey; ^16^ Department of Pathology and Laboratory Medicine, Department of Surgery (Urology) Brown University Warren Alpert Medical School, the Legorreta Cancer Center at Brown University, and Brown University Health Providence Rhode Island USA; ^17^ Department of Pathology, School of Medicine Atlas University Istanbul Turkey; ^18^ Department of Pathology Brigham and Women's Hospital Boston Massachusetts USA

**Keywords:** e‐cadherin, HER2, immunohistochemistry, p120 catenin, p63, plasmacytoid urothelial carcinoma, TRPS1

## Abstract

**Aims:**

Plasmacytoid urothelial carcinoma (PUC) is an aggressive morphological subtype characterized by discohesive single‐cell infiltration and frequent loss of epithelial adhesion. Although p63 is widely regarded as a reliable urothelial lineage marker in conventional urothelial carcinoma, its expression may be reduced or absent in plasmacytoid tumours, creating an important diagnostic pitfall. This multi‐institutional study aimed to characterize the clinicopathological and immunohistochemical profile of PUC, with particular emphasis on p63 immunoreactivity and its relationship to adhesion‐related markers and other diagnostically relevant immunophenotypes.

**Methods and results:**

A total of 93 cases initially diagnosed as PUC were retrospectively reviewed across multiple centres. After histopathological re‐evaluation, 70 confirmed cases were included. Immunohistochemical analysis was performed for p63, e‐cadherin, p120 catenin, TRPS1, *HER2/Neu*. The cohort comprised 70 patients with a median age of 66.5 years (IQR, 59.8–72.0) and a strong male predominance of 85.7% (60/70). Most specimens were obtained from transurethral resections (64.3%, 45/70) and radical cystectomy specimens (34.3%, 24/70). The median tumour size was 3.0 cm (IQR, 1.0–5.0), and the median proportion of plasmacytoid differentiation was 80% (IQR, 20–100). Morphologically, tumours were classified as classic plasmacytoid in 65.7% (46/70), pleomorphic in 22.9% (16/70) and desmoplastic in 11.4% (8/70). Concurrent variant histology was identified in 21.4% (15/70), most commonly micropapillary (12.9%, 9/70) and sarcomatoid (7.1%, 5/70) components. Lymphovascular invasion was present in 51.4% (36/70), and concomitant carcinoma in situ was detected in 35.7% (25/70). Immunohistochemically, p63 expression was retained in only 10.0% of tumours (7/70). HER2 membranous overexpression was observed in 58.6% (41/70), while loss of E‐cadherin expression was highly prevalent (84.3%, 59/70). Aberrant p120 catenin expression was common, with cytoplasmic localization in 72.9% (51/70) and complete loss in 17.1% (12/70). TRPS1 immunostaining was available in 54 tumours and was negative in 92.6% of evaluable cases (50/54).

**Conclusion:**

PUC demonstrates a distinctive immunophenotype characterized by frequent loss of p63 and disruption of the e‐cadherin/p120 adhesion complex. Recognition of p63 attenuation, together with adhesion‐related markers and HER2 status, provides a useful diagnostic framework for distinguishing this aggressive subtype from conventional urothelial carcinoma and its mimickers.

## Graphical abstract

Plasmacytoid urothelial carcinoma demonstrates marked attenuation of p63 expression together with frequent disruption of the E‐cadherin–p120 adhesion complex, supporting its characteristic discohesive growth pattern. Standardized HER2 scoring identifies a substantial positive subset, highlighting diagnostic pitfalls and potential therapeutic relevance in this aggressive subtype.

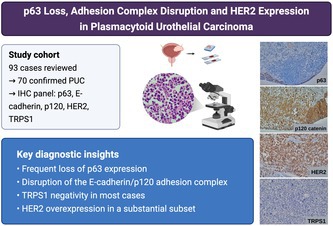

**Abbreviations**
ER: oestrogen receptor
H&E: haematoxylin and eosin
IQR: interquartile ranges
PR: progesterone receptor
PUC: plasmacytoid urothelial carcinoma

## Introduction

Plasmacytoid urothelial carcinoma (PUC) is an aggressive histologic subtype of urothelial carcinoma, characterized by diffuse growth or single‐cell infiltration of discohesive tumour cells, with or without cytoplasmic lumina or vacuoles. These tumours can spread extensively along tissue planes and peritoneal surfaces and are frequently associated with surgically occult involvement, underscoring the importance of careful recognition, particularly at resection margins.^1^, ^2^, ^3^

At the molecular and immunophenotypic level, plasmacytoid differentiation is closely linked to disruption of the cadherin–catenin adhesion complex. Frequent somatic loss‐of‐function alterations in *CDH1* provide a biologic basis for the characteristic loss of membranous e‐cadherin expression and the resulting discohesive architecture.^4^ In this context, immunohistochemical evaluation of e‐cadherin together with p120 catenin has emerged as a particularly useful diagnostic approach, as abnormal cytoplasmic p120 staining often parallels E‐cadherin loss and helps distinguish PUC from conventional urothelial carcinoma.^5^, ^6^ In addition, HER2/Neu overexpression and *HER2/Neu* amplification have been reported in a subset of plasmacytoid tumours, suggesting potential therapeutic relevance in selected patients.^7^

Despite these advances, an important and underrecognized diagnostic challenge in PUC is the altered behaviour of traditional urothelial lineage markers. In routine practice, p63 is widely regarded as a reliable marker of urothelial differentiation; however, plasmacytoid tumours may show reduced or complete loss of p63 expression, creating significant diagnostic pitfalls, particularly in limited biopsies and in the differential diagnosis with metastatic discohesive malignancies such as lobular breast carcinoma.^8^, ^9^, ^10^ Collectively, these observations highlight the need for an integrated immunophenotypic framework that emphasizes p63 loss while incorporating adhesion‐related markers and therapeutically relevant targets to refine the diagnostic landscape of this aggressive urothelial carcinoma subtype.

Therefore, in this multi‐institutional study, we aimed to characterize the expression patterns of p63, E‐cadherin, p120 catenin, trichorhinophalangeal syndrome 1 (TRPS1) and HER2/Neu in PUC, with particular emphasis on the diagnostic significance of p63 attenuation or loss in this distinctive subtype.

## Materials and Methods

### Study Design and Case Selection

This retrospective, multi‐institutional study included patients diagnosed with plasmacytoid urothelial carcinoma of the urinary tract. All available cases were identified from the pathology archives of the participating centres. Overall, 93 cases initially diagnosed as plasmacytoid urothelial carcinoma were collected. Additional central histopathology review (BYB and MA) was conducted; 23 cases were excluded due to the absence of true plasmacytoid morphology, yielding a final study cohort of 70 confirmed cases. The study protocol was approved by the Local Ethical Committee of Non‐invasive Clinical Research (GOKAEK‐2025/25/12).

**Figure 1:**
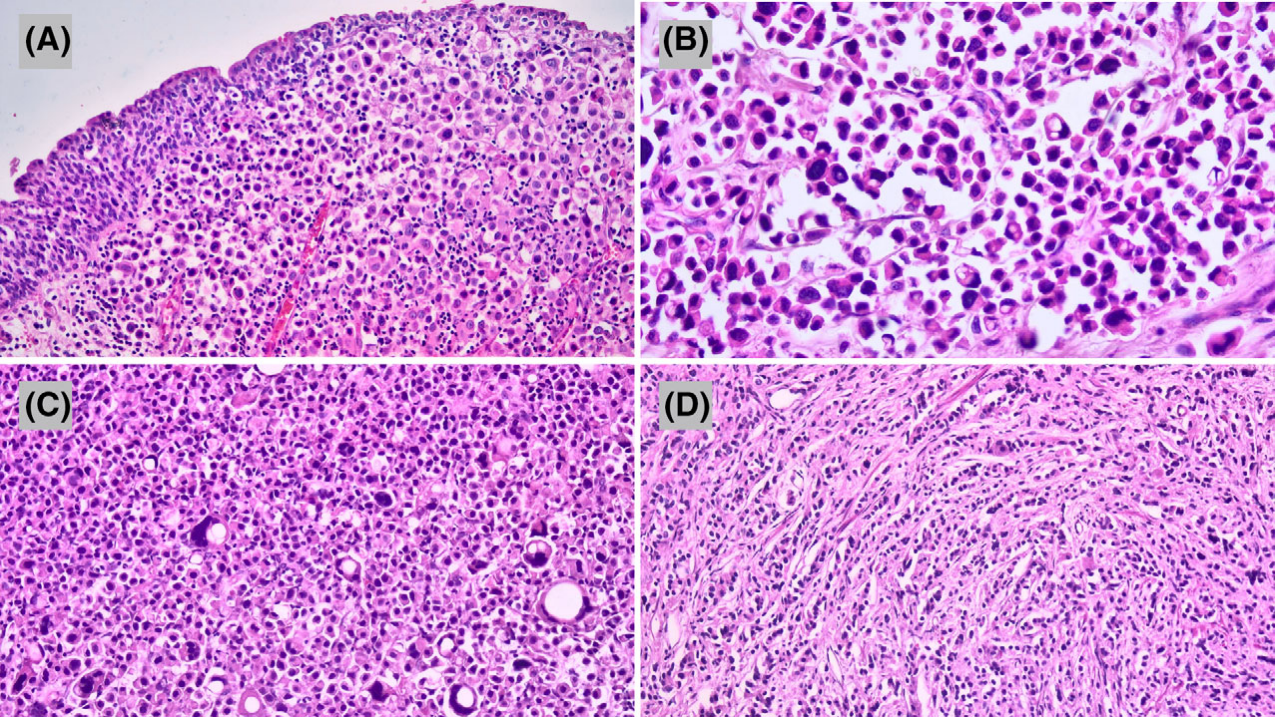
Morphologic spectrum of plasmacytoid urothelial carcinoma. (**A**) Classic plasmacytoid type composed of small discohesive tumour cells with eccentrically placed nuclei. (**B**) Plasmacytoid carcinoma with well‐formed signet ring–like cells in the absence of extracellular mucin. (**C**) Pleomorphic type showing enlarged, irregular and markedly atypical nuclei. (**D**) Desmoplastic type characterized by infiltrative tumour cells accompanied by prominent stromal desmoplasia.

Haematoxylin and eosin (H&E)–stained slides were reviewed by two pathologists (BYB and MA) to confirm PUC and to exclude cases that did not meet diagnostic criteria. Cases were included only when there was unequivocal plasmacytoid morphology, characterized by a discohesive infiltrative growth pattern composed of single cells or small clusters with eccentric nuclei and abundant eosinophilic cytoplasm, often mimicking plasma cells. Tumours with signet ring–like features in the absence of extracellular mucin were also considered within the plasmacytoid spectrum, in accordance with the current WHO classification.^3^, ^11^, ^12^ For further characterization, PUC were stratified into morphologic categories according to the classification scheme described by Perrino *et al*. and subsequently applied in comparative studies.^2^ Briefly, the *classic* type was defined by small discohesive tumour cells with eccentric nuclei (Figure 1A), sometimes including well‐formed signet ring–like cells (Figure 1B). The *pleomorphic* type was characterized by tumour cells showing enlarged, irregular and markedly atypical nuclei (Figure 1C). In addition, a *desmoplastic* type was recognized in cases demonstrating infiltrative tumour cells accompanied by prominent stromal desmoplasia (Figure 1D).^5^

**Figure 2:**
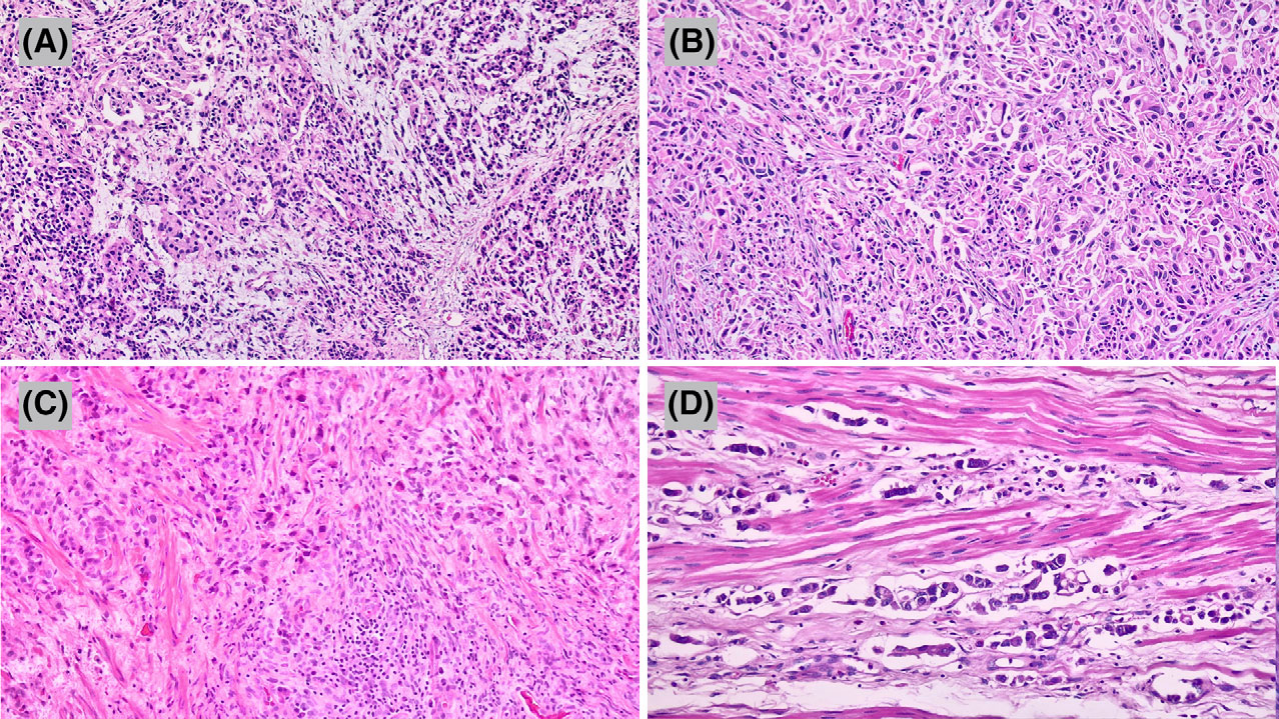
Diagnostic pitfalls and overlapping morphologic patterns that may mimic plasmacytoid urothelial carcinoma. (**A**) Urothelial carcinoma with myxoid and chordoid features demonstrating cord‐like arrangements within mucin‐like stromal material. (**B**) Areas of squamous differentiation that may be misinterpreted as plasmacytoid morphology. (**C**) Prominent desmoplasia complicating distinction from sarcomatoid differentiation. (**D**) Transurethral resection specimen showing crush/thermal artefact producing an artificial discohesive or ‘Indian‐file’ appearance.

In cases without an obvious conventional urothelial carcinoma component, additional morphologic review was performed to exclude diagnostic pitfalls related to overlapping variant patterns. These included tumours showing cord‐like arrangements within mucin‐like stromal material which are recently identified as ‘urothelial carcinoma with myxoid and chordoid features’ (Figure 2A),^13^ areas of squamous differentiation that could mimic plasmacytoid features (Figure 2B), and cases with prominent desmoplasia where distinction from sarcomatoid differentiation became challenging (Figure 2C). Furthermore, transurethral resection specimens with marked crush or thermal artefact occasionally created an artificial discohesive or ‘Indian‐file’ appearance (Figure 2D), leading to overinterpretation as PUC. Rare pleomorphic tumours with trophoblastic‐like features were also carefully evaluated to avoid misclassification as pleomorphic PUC.

Clinicopathological information was collected from pathology reports and medical records, including patient demographics, specimen type, tumour size, presence of concurrent variant histology or divergent differentiation, lymphovascular invasion, concomitant carcinoma in situ, TNM stage, margin status, metastatic involvement, follow‐up duration and survival status. Continuous variables were summarized as medians with interquartile ranges (IQR), and categorical variables were presented as counts and percentages. The presence of lymphovascular invasion was determined based on routine histopathological evaluation of haematoxylin and eosin–stained sections by the reviewing pathologists. Additional endothelial immunohistochemical stains such as CD31 or D2‐40 were not routinely performed across all participating centres and therefore were not systematically included in this retrospective analysis.

### Immunohistochemical Analysis

The immunohistochemical panel included: p63 (clone BC4A4, Dako, Santa Clara, CA), e‐cadherin (clone M3612, Dako Agilent, Santa Clara, California), p120 catenin (clone 98/pP‐120, BD Biosciences, San Jose, California), TRPS1 (clone PA5‐84874, ThermoFisher, Waltham, MA), HER2/Neu (clone SP3, Cell Marque, Rocklin, CA).

Marker selection was based on previously published studies evaluating immunophenotypic patterns in PUC and related differential diagnostic settings. Staining was evaluated according to the expected subcellular localization of each marker (nuclear, membranous, or cytoplasmic). p63 was recorded as positive or negative based on nuclear staining in tumour cells. E‐cadherin was scored as retained or lost according to the membranous expression in tumour cells. p120 catenin staining was categorized as membranous (normal), cytoplasmic, or negative (aberrant). TRPS1 expression was assessed as nuclear positive or negative. HER2/Neu was evaluated using ToGA‐based criteria (0–3+), and tumours showing membranous staining with a score of ≥2+ (moderate to strong complete or basolateral membranous staining) were classified as positive.^14^ Staining proportion and intensity were documented using a semi‐quantitative approach. All available immunohistochemical slides were reviewed in conjunction with the histological re‐evaluation during central review (MA, BYB). Nuclear p63 staining in tumour cells was recorded as positive irrespective of staining intensity, provided that unequivocal nuclear labelling was present. Partial loss of E‐cadherin expression was documented when heterogeneous membranous staining was observed within tumour cell populations. In cases demonstrating E‐cadherin loss, the pattern of p120 catenin expression was evaluated to identify the characteristic cytoplasmic redistribution associated with disruption of the adhesion complex.

## Results

### Clinicopathological Characteristics of the Study Cohort

The clinicopathological characteristics of the 70 confirmed cases are summarized in Table 1. The median age of the patients was 66.5 years (IQR, 59.8–72.0), with a marked male predominance (85.7%, 60/70). Most patients were symptomatic at presentation (75.7%, 53/70). Specimens were obtained predominantly from transurethral resections (64.3%, 45/70) and radical cystectomy samples (34.3%, 24/70) (Table 1).

**Table 1:** Clinical findings of all patients with plasmacytoid urothelial carcinoma (*n* = 70)

Variable	All cases (*n* = 70)
Age, years (median [IQR])	66.5 [59.8–72.0]
Gender, *n* (%)	
Male	60 (85.7)
Female	10 (14.3)
Presenting symptoms, *n* (%)	
Asymptomatic	17 (24.3)
Symptomatic	53 (75.7)
Specimen type, *n* (%)	
Biopsy	1 (1.4)
Transurethral resection	45 (64.3)
Radical cystectomy	24 (34.3)
Follow‐up, months (median [IQR])	11 [7–25]
Death during follow‐up, *n* (%)	35 (50)

The median tumour size was 3.0 cm (IQR, 1.0–5.0), and the median proportion of plasmacytoid differentiation was 80% (IQR, 20–100). Histologically, the classic plasmacytoid pattern was the most frequent subtype (65.7%, 46/70), whereas pleomorphic PUC accounted for 22.9% (16/70) of cases. A desmoplastic subtype was identified in 11.4% (8/70) of tumours (Table 2).

**Table 2:** Pathological characteristics of all patients with plasmacytoid urothelial carcinoma (*n* = 70)

Variable	All cases (*n* = 70)
Tumour size, cm (median [IQR])	3.0 [1.0–5.0]
Plasmacytoid subtype %, median [IQR]	80 [20–100]
Plasmacytoid category, *n* (%)	
Classic	46 (65.7)
Pleomorphic	16 (22.9)
Desmoplastic	8 (11.4)
Concurrent subtype histology, *n* (%)	
Micropapillary	9 (12.9)
Sarcomatoid	5 (7.1)
Giant cell	1 (1.4)
Lipid‐rich	1 (1.4)
Poorly differentiated	2 (2.9)
Concurrent divergent differentiation, *n* (%)	
Squamous	7 (10)
Glandular	9 (12.9)
Trophoblastic	3 (4.3)
Lymphovascular invasion, *n* (%)	36 (51.4)
Concomitant CIS present, *n* (%)	25 (35.7)
pT category, *n* (%)	
pT1	14 (20)
pT2	32 (45.7)
pT3a	6 (8.6)
pT3b	4 (5.7)
pT4	14 (20)
pN category, *n* (%)	
Nx	27 (38.6)
N0	24 (34.3)
N1	13 (18.6)
N2	6 (8.6)
pM category, *n* (%)	
Mx	34 (48.6)
M0	26 (37.1)
M1	10 (14.3)
Another organ involvement, *n* (%)	27 (38.6)
Primary IHC outcomes, *n* (%)	
p63 positive, *n* (%)	7 (10)
p63 nuclear staining (% positive tumour cells, median [IQR])	0 [0–100]
HER2 membranous overexpression, *n* (%)	41 (58.6)
E‐cadherin lost, *n* (%)	59 (84.3)
p120 cytoplasmic, *n* (%)	51 (72.9)
p120 negative, *n* (%)	12 (17.1)
TRPS1 negative, *n* (%)	50 (of 54 patients)

CIS, carcinoma in situ; IHC, immunohistochemistry.

Concurrent histological subtypes were identified in a subset of tumours, most commonly with a micropapillary component (12.9%, 9/70) and sarcomatoid differentiation (7.1%, 5/70). Other concurrent subtype morphologies included giant cell (1.4%, 1/70), lipid‐rich (1.4%, 1/70) and poorly differentiated (2.9%, 2/70). Divergent differentiation was also observed, including glandular (12.9%, 9/70), squamous (10.0%, 7/70) and trophoblastic differentiation (4.3%, 3/70).

Lymphovascular invasion was present in 51.4% (36/70) of patients, and concomitant carcinoma in situ was detected in 35.7% (25/70). Pathologic staging showed that most tumours were muscle invasive at diagnosis, with pT2 disease in 45.7% (32/70) and pT4 disease in 20.0% (14/70). Additional staging categories included pT1 in 20.0% (14/70), pT3a in 8.6% (6/70) and pT3b in 5.7% (4/70).

Among the cohort, nodal status was pN0 in 34.3% (24/70), pN1 in 18.6% (13/70) and pN2 in 8.6% (6/70). Distant metastatic disease was identified in 14.3% (10/70) of patients (pM1) (Table 2).

In addition, other organ involvement was observed in 38.6% (27/70) of cases, reflecting the aggressive and infiltrative behaviour of PUC. Among the 24 patients who underwent cystectomy, positive surgical margins were identified in 7 cases (29.2%), including ureteral margin positivity in 6 cases (25.0%) and urethral margin positivity in 1 case (4.2%).

The median follow‐up duration was 11 months (IQR, 7–25), and overall mortality was 50.0% (35/70).

### Immunohistochemical Profile

**Figure 3:**
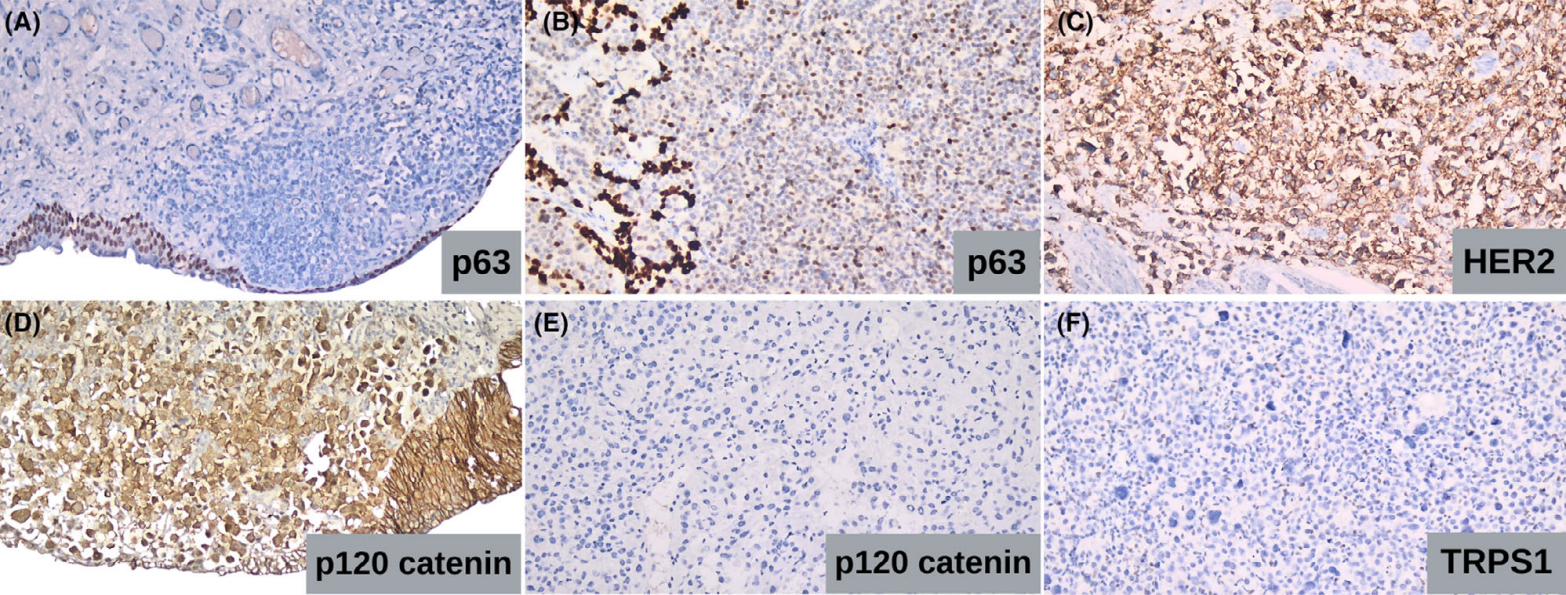
Immunohistochemical profile of plasmacytoid urothelial carcinoma demonstrating attenuation of basal urothelial markers and disruption of epithelial adhesion pathways. (**A**) Loss of p63 nuclear expression in tumour cells. (**B**) Retained nuclear p63 staining in a rare p63‐positive plasmacytoid carcinoma. (**C**) HER2 membranous positivity. (**D**) Aberrant cytoplasmic localization of p120 catenin. (**E**) Complete loss of p120 catenin staining. (**F**) Negative TRPS1 staining in tumour cells. [Colour figure can be viewed at wileyonlinelibrary.com]

Immunohistochemical evaluation highlighted a characteristic profile for PUC (Table 2). Overall, p63 expression was absent in 90.0% of tumours (63/70) (Figure 3A), whereas retained nuclear p63 staining was identified in only 10.0% (7/70) (Figure 3B), supporting the well‐recognized attenuation of basal urothelial markers in this aggressive variant.

Among the p63‐negative tumours (63/70, 90.0%), 61.9% were classified as classic plasmacytoid (39/63), while 25.4% showed pleomorphic morphology (16/63) and 12.7% demonstrated a desmoplastic subtype (8/63). All p63‐positive cases belonged to the classic plasmacytoid category (7/7, 100%). Comparison between p63‐positive and p63‐negative tumours revealed several notable trends. HER2 membranous overexpression was observed in 5 of the 7 p63‐positive tumours (71.4%). TRPS1 staining was performed in four of the seven p63‐positive cases and was negative in three of these cases (3/4, 75.0%). TRPS1 expression was rare overall and did not show a consistent association with p63 status.

HER2 membranous overexpression (≥2+) was observed in 58.6% (41/70) (Figure 3C), while loss of e‐cadherin expression was highly prevalent in 84.3% (59/70), consistent with the characteristic discohesive growth pattern of PUC. Aberrant p120 catenin expression was common, with cytoplasmic localization observed in 72.9% (51/70) (Figure 3D), while complete loss of p120 staining was identified in 17.1% (12/70) (Figure 3E), supporting disruption of the e‐cadherin–catenin adhesion complex.

TRPS1 was negative in 92.6% of evaluated tumours (50/54) (Figure 3F), while only 4/54 (7.4%) showed any nuclear positivity, which was typically focal and weak (Table 2).

## Discussion

In this multi‐institutional study, we comprehensively characterized the morphologic and immunohistochemical landscape of PUC. One of the most diagnostically relevant observations in our cohort was the near‐universal loss of p63 expression, with only a small minority of tumours showing retained nuclear staining. p63 expression is typically preserved in the vast majority of conventional urothelial carcinomas, with reported positivity rates of approximately 90%–95%, supporting its role as a robust urothelial lineage marker.^8^, ^11^, ^15^, ^16^ While p63 is routinely used to support urothelial differentiation in conventional urothelial carcinoma,^2^, ^6^, ^8^, ^11^, ^12^ its reduced expression in plasmacytoid tumours represents a diagnostic challenge, particularly in small biopsies or metastatic settings.^2^, ^6^, ^17^, ^18^ Paner *et al*. previously demonstrated that urothelial differentiation markers may show variable performance across histological subtypes, emphasizing the need for careful interpretation in nonconventional subtypes.^8^ In this context, our findings reinforce that p63 negativity should not exclude a urothelial primary, especially when plasmacytoid morphology is present and that diagnostic evaluation must incorporate both morphology and an expanded immunohistochemical framework.

Beyond diagnostic markers, our study also demonstrated HER2 membranous overexpression in more than half of the evaluated tumours. This finding aligns with prior work by Kim *et al*., who reported HER2 protein overexpression and *HER2* amplification in PUC, raising the possibility of targeted therapeutic strategies in selected patients.^7^ Importantly, HER2 was interpreted using established ToGA‐based gastric cancer scoring criteria (0–3+), and tumours with a score of ≥2+ membranous staining were regarded as positive.^14^ This standardized approach may improve inter‐institutional consistency and supports the emerging concept that HER2 may represent a therapeutically relevant target in PUC, particularly in the era of antibody–drug conjugates. Future studies integrating HER2 IHC with confirmatory amplification testing and clinical response data will be essential to clarify its predictive significance.

TRPS1 has emerged as a highly sensitive marker for breast carcinoma, particularly in triple‐negative settings, as demonstrated by Ai *et al*.^19^ and large‐scale tumour profiling studies.^19^, ^20^ However, its role in PUC remains evolving. In our cohort, TRPS1 was frequently negative, indicating that it is not a reliable marker for confirming urothelial lineage, but rather may serve as a useful exclusionary marker in the differential diagnosis with metastatic lobular breast carcinoma, where TRPS1 expression is typically retained. This issue becomes especially important in the differential diagnosis between PUC and metastatic lobular breast carcinoma involving the bladder, a scenario highlighted by Borhan *et al*.^9^ and further explored in detail by Zhang *et al*.^10^ Given the overlapping discohesive morphology, reliance on a single marker may be misleading, and comprehensive panels incorporating urothelial, adhesion and breast‐associated markers remain critical. Recent work by Bachert *et al*.^21^ has also questioned the absolute specificity of TRPS1, demonstrating expression in a subset of non‐breast malignancies. In the differential diagnosis with metastatic lobular breast carcinoma involving the bladder, additional breast‐associated markers such as oestrogen receptor (ER) and progesterone receptor (PR) may also be informative. Most lobular breast carcinomas show ER and PR positivity and are typically HER2‐negative, whereas plasmacytoid urothelial carcinoma generally lacks consistent hormone receptor expression. Although rare cases of PUC may demonstrate focal PR staining, the combined interpretation of morphology together with a broader immunohistochemical panel (including urothelial, adhesion‐related and breast‐associated markers) usually allows reliable distinction between these entities in routine diagnostic practice.^9^, ^10^

Consistent with prior clinical series and systematic reviews,^1^, ^22^, ^23^, ^24^, ^25^, ^26^, ^27^ PUC in our study frequently presented at advanced stage and was associated with substantial mortality. Kaimakliotis *et al*. previously described the distinctive invasive pattern of PUC along fascial planes, providing an anatomic basis for its propensity towards extensive spread and understaging.^1^ Several outcome‐based studies have further emphasized the aggressive course of this subtype, including meta‐analytic evidence from Kim *et al*.^22^ and treatment‐response evaluations by Diamantopoulos *et al*.^26^ and Sorce *et al*.^28^ Importantly, Davaro *et al*. demonstrated that surgical margin status and extent of lymphadenectomy may significantly impact oncologic outcomes, underscoring the importance of meticulous surgical management.^27^ Our findings of frequent other organ involvement and occasional positive margins further support the need for heightened clinical awareness and multidisciplinary planning when managing patients with PUC.^29^

This study has several limitations inherent to its retrospective multi‐institutional design. Although our cohort represents one of the larger clinicopathological series of plasmacytoid urothelial carcinoma with detailed morphologic review and immunohistochemical characterization, variability in available clinical follow‐up data across participating centers limited the ability to perform more uniform outcome‐based analyses. In addition, not all immunohistochemical markers were available in every case, reflecting differences in archival material and institutional testing practices. Future investigations should focus on integrating immunophenotypic findings with next‐generation sequencing data, immune microenvironment profiling and therapeutic response assessment, as emerging molecular studies have demonstrated substantial genomic heterogeneity across aggressive urothelial carcinoma subtypes.^22^, ^30^, ^31^, ^32^ Such approaches may help refine biomarker‐driven diagnostic and treatment algorithms for plasmacytoid urothelial carcinoma.

From a practical diagnostic perspective, recognition of the characteristic immunophenotypic profile of PUC is particularly important in limited biopsy or transurethral resection specimens. In such settings, reduced or absent p63 expression may initially raise concern for a non‐urothelial malignancy. Awareness that plasmacytoid tumours frequently demonstrate attenuation of p63, together with evaluation of E‐cadherin and p120 catenin, can help prevent diagnostic misinterpretation and guide the appropriate use of additional markers when necessary.

In conclusion, plasmacytoid urothelial carcinoma demonstrates a distinctive clinicopathological and immunophenotypic profile characterized by frequent attenuation of p63 expression and marked disruption of the E‐cadherin–p120 adhesion complex, providing an important diagnostic framework for this aggressive variant. The high prevalence of HER2 membranous overexpression further suggests potential therapeutic relevance in selected patients. Although TRPS1 is typically absent in PUC, it remains valuable within a broader immunohistochemical panel for distinguishing PUC from metastatic mimickers such as lobular breast carcinoma. Recognition of these features is essential for accurate diagnosis, appropriate clinical management and future biomarker‐driven studies in this challenging subtype.

## Author contributions

All authors collected the data. BYB, MA drafted the manuscript. BYB, KK, YYK, LC and MA edited the manuscript, participated in the study design and coordination. All authors read and approved of the final manuscript.

## Funding information

The authors have no financial disclosures relevant to this work.

## Conflicts of interest

The authors declare that they have no competing interests.

## Data Availability

All data generated or analysed during this study were included in this published article.
